# Severe Drug-Induced Agranulocytosis Successfully Treated with Recombinant Human Granulocyte Colony-Stimulating Factor

**DOI:** 10.1155/2018/8439791

**Published:** 2018-01-23

**Authors:** Yohei Kubota, Toh Yoon Ezekiel Wong

**Affiliations:** Department of Internal Medicine, Hiroshima Kyoritsu Hospital, Hiroshima, Japan

## Abstract

When elderly patients are prescribed many different medications, the risk for developing serious adverse events should be kept in mind. One of these adverse events is agranulocytosis, which, although rare, can be life-threatening if left untreated. The majority of agranulocytosis cases are caused by drugs, including antibiotics. Here, we report a case of severe agranulocytosis in a 96-year-old woman following antibiotic therapy which was successfully managed using recombinant human granulocyte colony-stimulating factor (rhG-CSF) and the appropriate choice of antibiotics to treat her concomitant infection.

## 1. Background

Agranulocytosis is a rare and life-threatening condition, with an annual incidence of 1.1 to 4.9 cases per million population per year [1]. Up to 50% of agranulocytosis may be induced by nonchemotherapy drugs, such as antithyroid agents and antimicrobial medications [2]. The risk for developing this adverse event may be higher among elderly patients in Japan, who are often prescribed many types of medication, a phenomenon called polypharmacy [3].

The problem of polypharmacy is of particular concern in an aging society like Japan because elderly patients tend to have many underlying diseases as well as conditions that require various medications. We herein present a case of severe agranulocytosis following antibiotic treatment with garenoxacin and ceftriaxone. The patient was also prescribed 10 different kinds of medication regularly, making this a case that emphasizes the importance of recognizing severe drug-related adverse events and the potential danger of polypharmacy or unnecessary prescription of antibiotics.

## 2. Case Presentation

A 96-year-old woman was prescribed garenoxacin for 8 days after complaining of a bad cough, dyspnea, and anorexia at a nearby clinic. This was despite a simple laboratory blood test revealing a white blood cell (WBC) count of 7920/*μ*L (55% neutrophils) and a C-reactive protein (CRP) level of 0.33 mg/dL, findings that were unremarkable. However, she developed high-grade fever on the following day, and intravenous ceftriaxone (1 g daily for 6 days) was added to her treatment regime two days after prescribing garenoxacin. After 5 days of therapy with ceftriaxone, a follow-up laboratory blood test performed showed a WBC count of 1050/*μ*L (0% neutrophils) and a CRP level of 6.67 mg/dL. Severe leucopenia was suspected, and she was referred to our hospital for evaluation.

During presentation, her blood pressure was 136/99 mmHg, her pulse rate was 87 beats per minute, and her body temperature was 37.4°C. Her respiratory rate was 20 breaths per minute and peripheral oxygen saturation 97%. Chest sounds were normal, and other findings of physical examination were unremarkable. She was 150 cm in height with mild kyphosis and weighed 40 kg. She had a medical history of atrial fibrillation, stroke, hypertension, hypotonic urinary bladder, lower back pain, and gastritis. Her daily medications included candesartan, lansoprazole, amlodipine, carbocysteine, zaltoprofen, tranexamic acid, rivaroxaban, bisoprolol, brotizolam, and celecoxib.

Full blood count performed at our hospital revealed severe leucopenia with a WBC count of 830/*µ*L (neutrophils 7/*µ*L, eosinophils 8/*μ*L, basophils 18/*μ*L, monocytes 378/*μ*L, and lymphocytes 415/*μ*L). Other laboratory data are shown in Table 1. A chest radiograph and computed tomography showed mild infiltrative shadow on her left lung. However, both blood and sputum culture results were negative.

She was diagnosed with drug-induced agranulocytosis (presumably from antibiotics used) with mild pneumonia and admitted to an isolated room. Candesartan, lansoprazole, amlodipine, and celecoxib were discontinued because they are also known to cause agranulocytosis. She was given meropenem hydrate intravenously (2 g/day) and levofloxacin hydrate orally (500 mg/day) for 10 days, after which a short course of sulfamethoxazole/trimethoprim (4 g/day) was administered. Subcutaneous injection of filgrastim (recombinant human G-CSF, 75 *µ*g/day) was also given to treat her neutropenia.

Filgrastim was administered for 6 days before the neutrophil count normalized to 2539/*µ*L on day 6. Although her neutrophil count increased to 12,940/*µ*L on day 9, it eventually stabilized around 3000 to 4000/*µ*L after day 13.  Table 2 summarizes the changes in WBC count, neutrophil count, and CRP levels during her hospitalization. Her fever receded after day 10, and she was discharged on day 18 without further complications.

## 3. Discussion

Although there is no clear consensus, polypharmacy may be defined as the concomitant use of five or more medications per day [4]. Polypharmacy is a major concern in Japan as older patients such as ours are often given many different medications (10 in our patient) on a regular basis. The fact that patients have easy access to different specialists in Japan also increases the probability of polypharmacy as they often see many physicians without a primary care doctor carefully monitoring their medications. Furthermore, our patient was prescribed antibiotics, which may be due to her old age and the risk of developing pneumonia, after presenting with symptoms of upper respiratory tract infection. The questionable practice of treating acute respiratory infections with antibiotics is still common in Japan. Lack of proper patient education has resulted in increased patient satisfaction when they are prescribed antibiotics [5]. Studies have also demonstrated that the use of antibiotics for acute respiratory infections does not result in increased adverse events and may even be slightly beneficial in preventing pneumonia hospitalization [6].

Nevertheless, a recent study showed that older female patients as well as those with multiple comorbidities and medications (which fit our patient's profile) have the highest risk of adverse drug reactions in the acute care setting [7]. This includes life-threatening conditions such as agranulocytosis. Agranulocytosis, although rare, can be caused by many different drugs, including antibiotics [2, 8]. Without early detection, this condition may result in serious infections and subsequently sepsis [9]. In our patient's case, due to the timing and development of her agranulocytosis, we believe that it was most probably caused by garenoxacin or ceftriaxone, either directly or through interactions with her other drugs.

Currently, the standard treatment for drug-induced agranulocytosis is discontinuation of the responsible agent(s), antibiotic treatment if the presence of infection is suspected, and proper use of granulocyte colony-stimulating factor (G-CSF). Although their efficacy is not conclusively proven, G-CSF has minimal toxicity and may be beneficial in the management of drug-induced agranulocytosis in elderly patients [10]. Minor complications include bone pain and leukocytosis, which occurred in our patient but normalized after discontinuing therapy. As for antibiotic treatment, cefepime (a fourth-generation cephalosporin) is often employed as a first-line therapy for agranulocytosis with infection. Meropenem can be used as an alternative when cefepime is not tolerated well [11]. A recent study also demonstrated that, for severe cases (neutrophil count < 100/*µ*L), meropenem may be the superior choice for monotherapy [12]. Meropenem was used due to the severely low neutrophil count and also because our patient's agranulocytosis may have been caused by ceftriaxone (a third-generation cephalosporin).

In conclusion, we reported a case of severe agranulocytosis in an elderly patient which was managed successfully using rhG-CSF and proper choice of antibiotics. Elderly patients who are being prescribed many different medications need to be carefully managed and monitored for various adverse drug events.

## Figures and Tables

**Table 1:** Laboratory data on admission.

WBC	830	/*μ*L	TP	6.1	g/dL
(i) Neutrophil	0.9	%	Alb	2.8	g/dL
(ii) Lymphocyte	50.2	%	T-Bil	0.4	mg/dL
(iii) Basophil	2.2	%	AST	19	U/L
(iv) Eosinophil	1.0	%	ALT	15	U/L
(v) Monocyte	45.8	%	LDH	144	U/L
RBC	345	×10^4^/*μ*L	ALP	185	U/L
Hb	10.3	g/dL	γ-GTP	15	U/L
Ht	32.0	%	AMY	63	U/L
Plt	10.2	×10^4^/*μ*L	BUN	25.1	mg/dL
CRP	8.14	mg/dL	Cr	0.75	mg/dL
ABGA (O_2_ 10 L/min by reservoir mask)			Na	137	mEq/L
pH	7.390	g/dL	K	4.1	mEq/L
PaCO_2_	32.5	mmHg	Cl	108	mEq/L
PaO_2_	125.0	mmHg	Glucose	113	mg/dL
HCO_3_^−^	19.2	mmol/L	PT-INR	1.67	—
SaO_2_	98.9	%	APTT	44.5	*s*

WBC: white blood cell; RBC: red blood cell; Hb: hemoglobin; Ht: hematocrit; Plt: platelet; CRP: C-reactive protein; ABGA: arterial blood gas analysis; TP: total protein; Alb: albumin; T-Bil: total bilirubin; AMY: amylase; BUN: blood urea nitrogen; Cr: creatinine; PT-INR: international normalized ratio of prothrombin time; APTT: activated partial thromboplastin time.

**Table 2:** Change in laboratory data after admission.

Day	1	2	3	6	9	13
WBC (/*μ*L)	830	800	840	4660	15970	4910
Neutrophil (/*μ*L)	7	21	58	2539	12940	3358
CRP (mg/dL)	8.14	18.54	—	4.16	4.26	1.74

WBC: white blood cell; CRP: C-reactive protein.
