# Erratum to “Severe Drug-Induced Agranulocytosis Successfully Treated with Recombinant Human Granulocyte Colony-Stimulating Factor”

**DOI:** 10.1155/2018/6749432

**Published:** 2018-06-24

**Authors:** Yohei Kubota, Ezekiel Wong Toh Yoon

**Affiliations:** Department of Internal Medicine, Hiroshima Kyoritsu Hospital, Hiroshima, Japan

In the article titled “Severe Drug-Induced Agranulocytosis Successfully Treated with Recombinant Human Granulocyte Colony-Stimulating Factor” [1], the second author's first and last names were reversed. The corrected authors' list is shown above.
